# Matrix Metalloproteinase-9 (MMP-9) as a Cancer Biomarker and MMP-9 Biosensors: Recent Advances

**DOI:** 10.3390/s18103249

**Published:** 2018-09-27

**Authors:** Hao Huang

**Affiliations:** 1 Department of Pediatric Oncology, Dana-Farber Cancer Institute, Boston, MA 02215, USA; hao_huang@dfci.harvard.edu; Tel.: +1-617-582-8046; 2 Department of Pediatrics, Harvard Medical School, Boston, MA 02115, USA

**Keywords:** matrix metalloproteinase-9, biomarker, cancers, biosensor, cancer detection, matrix metalloproteinases

## Abstract

As one of the most widely investigated matrix metalloproteinases (MMPs), MMP-9 is a significant protease which plays vital roles in many biological processes. MMP-9 can cleave many extracellular matrix (ECM) proteins to regulate ECM remodeling. It can also cleave many plasma surface proteins to release them from the cell surface. MMP-9 has been widely found to relate to the pathology of cancers, including but not limited to invasion, metastasis and angiogenesis. Some recent research evaluated the value of MMP-9 as biomarkers to various specific cancers. Besides, recent research of MMP-9 biosensors discovered various novel MMP-9 biosensors to detect this enzyme. In this review, some recent advances in exploring MMP-9 as a biomarker in different cancers are summarized, and recent discoveries of novel MMP-9 biosensors are also presented.

## 1. Introduction

### 1.1. Characteristic of Human MMP-9

MMPs are a family of zinc-dependent endopeptidases with more than 20 different members [1]. MMP family members can be organized into several groups, such as gelatinases, collagenases, stromelysins, matrilysins and membrane-type MMPs [2]. In particular, MMP-9, which plays vital roles in cancer cell invasion and tumor metastasis, is one of the most widely investigated MMPs. This review focuses on MMP-9, although it is not the only MMP member with vital roles in cancer development. Together with MMP-2, MMP-9 belongs to the gelatinase subgroup of the MMP family. Overexpression of MMP-9 has often been observed in different malignant tumors. Many studies have explored MMP-9 as a biomarker in different types of cancer. Additionally, various novel MMP-9 biosensors have been developed to detect this protein. In this review, we will describe some recent advances about exploring MMP-9 as a biomarker and MMP-9 biosensors.

**Figure 1:**
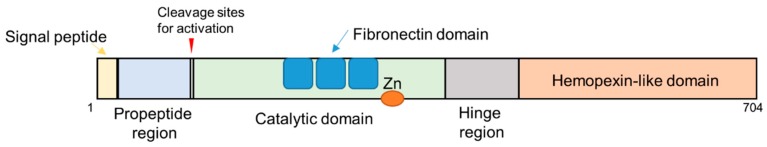
Schematic illustration of domain structures and motifs in MMP-9.

The human MMP-9 gene is located at chromosome 20q13.12. This gene contains 13 exons and 12 introns. Human MMP-9 protein contains hemopexin-like domain, catalytic domain, signal peptide, the hinge region and propeptide region (Figure 1) [2,3,4]. The catalytic domain of MMP-9 contains fibronectin type II (FN2) domains, active site and zinc-binding region. This protein contains two zinc ions and five calcium ions. As a member of zinc-dependent endopeptidases, MMP-9 requires zinc ion for its catalytic activity [3,5,6,7].

Although some studies have already revealed the structural information about some domains of MMP-9, the structure of the complete enzyme is still not known. One reason that the structure of the complete enzyme is difficult to reveal is that some regions of MMP-9 are highly flexible, such as some parts of the hinge region. The hinge region confers flexibility between hemopexin-like domain and catalytic domain to MMP-9, which may be critical to the function of this enzyme [8,9,10]. The catalytic domain of MMP-9 plays a critical role in the catalytic activity of this enzyme. Both active site and zinc-binding region within this domain are essential to enzyme activity. Additionally, the fibronectin domain is an important domain to MMP-9. This domain is important for some substrate binding and degradation [11,12,13].

The MMP-9 hemopexin-like domain consists of a four-bladed β-propeller to form a barrel-shaped structure [3,9]. The hemopexin-like domain of MMP-9 can interact with substrates, such as gelatin and collagen. This domain is important for specificity during substrate recognition [14]. As natural inhibitors of MMP-9, some tissue inhibitor of metalloproteinases (TIMPs), such as TIMP-1, can bind to the hemopexin-like domain of pro-MMP-9 to form a tight complex which can prevent the activation of MMP-9 [2,15,16,17]. The hemopexin-like domain of MMP-9 is relatively special, which may cause distinct biological roles of MMP-9 in cell function. The MMP-9 hemopexin-like domain forms a separate cluster during sequence alignment analysis of hemopexin-like domains of all human MMPs [18]. Additionally, it shares low amino acid identity with hemopexin-like domains from other MMPs [19].

In humans, some types of cells can synthesize and secrete MMP-9, which include neutrophils, macrophages, fibroblasts and endothelial cells [3]. MMP-9 is synthesized as a pre-proenzyme with 19 amino acid N-terminal signal peptides within cells. Then, it is secreted into the extracellular environment as inactive pro-MMP-9 (about 92 kDa). Activation of MMP-9 requires cleavage by other proteases after pro-MMP-9 is secreted [3,5,20]. Some proteases, such as MMP-3, can cleave inactive pro-MMP-9 to generate active MMP-9 (about 82 kDa) by removing the N-terminal propeptide region from pro-MMP-9 [21,22,23,24]. A cysteine switch is vital to the activation of MMP-9. One cysteine (Cys99) residue in the propeptide of pro-MMP-9 can interact with the catalytic zinc ion of this protein. This interaction is required in the maintenance of MMP latency. The proteolytic removal of the propeptide region will completely disrupt this interaction. After activation, MMP-9 can cleave its substrates at MMP-9 cleavage sites. In addition to proteolytic activation, some agents can also affect the activity of MMP-9, such as some reactive oxygen species (ROS) and nitric oxide [25,26,27,28,29]. ROS may perturb the interaction between the catalytic zinc ion and Cys99, which can cause MMP-9 activation.

Usually, to those MMP-9 substrates, the amino acid residue sequences surrounding the cleavage site determine whether this position can be efficiently cleaved by MMP-9 [30,31]. The most common consensus sequence of these cleavage sites contains sequence Pro-X-X-Hy-(Ser/Thr) at P3 position through P2′ position (X represents any residue; Hy represents a hydrophobic residue; cleavage site locates between P1-P1′) [30,31].MMP-9 was regulated by multiple levels of regulation, which include transcription, post-transcriptional regulation (miRNA regulation), translation, secretion of proMMP-9, post-translational regulation (proMMP-9 activation), and inhibition (TIMPs inhibition) [3,5,20,32,33,34,35,36,37].

**Figure 2:**
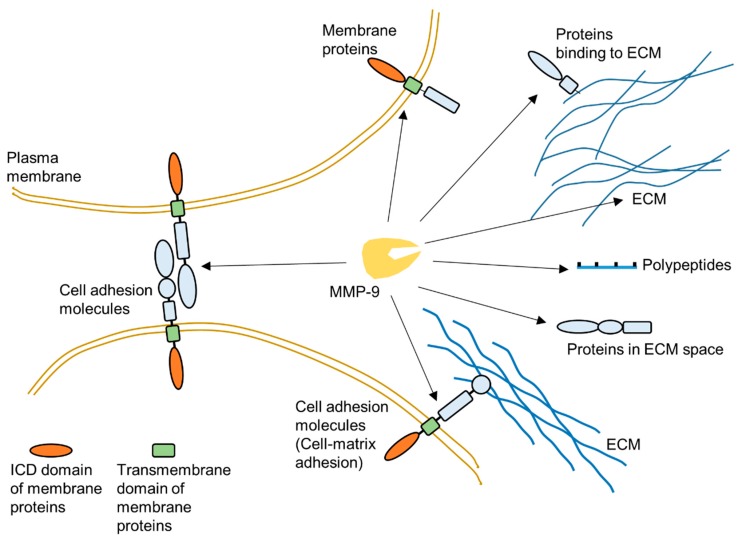
Schematic illustration about targets of MMP-9 in extracellular environment.

As one of the most widely investigated MMPs, MMP-9 is a significant protease which plays a vital role in many biological processes, such as wound healing [3,8,38,39,40,41,42,43]. This protease has a broad range of substrates. The important substrates of MMP-9 include, but are not limited to, gelatin, collagen and elastin. MMP-9 can degrade many extracellular matrix (ECM) proteins through proteolytic cleavage to regulate ECM remodeling (Figure 2) [3,20,44,45]. Additionally, some plasma membrane proteins are targets of MMP-9 [46,47]. MMP-9 can specifically cleave extracellular domain of some cell surface proteins to release them from the plasma membrane (Figure 2). In extracellular space, some polypeptides can also be cleaved by MMP-9 [39]. Moreover, MMP-9 could also cleave intracellular substrates after it was activated within cells [48,49,50,51,52,53,54,55]. Figure 2 summarizes some targets of MMP-9 in the extracellular space.

### 1.2. Biological Function of MMP-9

**Figure 3:**
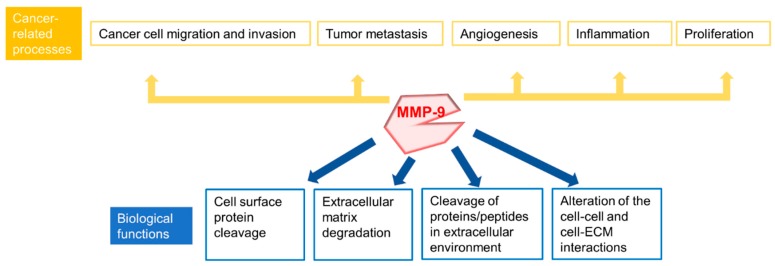
Summary of biological functions of MMP-9 and some cancer-related processes involved MMP-9.

Because of its proteolytic cleavage activity in the extracellular environment, MMP-9 is involved in many biological processes. These biological processes include, but are not limited to, proteolytic degradation of ECM, alteration of cell-cell and cell-ECM interactions, cleavage of cell surface proteins and cleavage of proteins in extracellular environment (Figure 3) [3,20,39,44,45,46,47,56,57,58,59,60,61,62]. MMP-9 plays a role in basement membrane degradation, since basement membrane contains collagens, including Type IV Collagen, which can be degraded by MMP-9 [56,57,58]. During tumor development, basement membrane destruction is usually an essential step which supports tumor invasion and metastases.

### 1.3. MMP-9 and Cancers

Several important processes of carcinogenesis, which include, but are not limited to, migration, invasion, metastasis, and angiogenesis, are closely related to the extracellular environment [63]. Because MMP-9 plays an important role in ECM remodeling and membrane protein cleavage, it is found to be widely associated with cancer pathologies (Figure 3) [63,64,65,66,67,68,69,70,71,72]. For instance, MMP-9 has been found to play a role in tumor invasion, metastasis and angiogenesis and to mediate tumor microenvironment [45,64,65,66,67,68,69,73,74,75]. MMP-9 promotes cancer development and progression in most cases, and it can also play a suppressive role in cancer progression in some specific cases, such as colitis-associated colon cancer [76,77,78]. In addition to cancers, MMP-9 is also associated with the pathologies of some other diseases, including autoimmune diseases and cardiovascular diseases, which will not be addressed in this review [17,79,80,81,82,83].

Since MMP-9 is an important target for several cancers and some other MMP-9 related diseases, targeting MMP-9 is of very high value. MMP-9 inhibitor development is an important research area to achieve this goal. To date, there has been no successful specific MMP-9 inhibitor utilized in the clinic. Due to the similarity among different MMPs, achieving the specificity of an MMP inhibitor (including MMP-9 inhibitor) to avoid off-target effect to other MMPs is a strong challenge to MMP inhibitor development. We will not introduce detailed information about MMP-9 inhibitor in this review. Much research has discovered the value of MMP-9 as a potential biomarker in various cancers [84,85,86,87,88]. In this review, we summarize some recent advances about exploring MMP-9 as a biomarker in different cancers.

## 2. MMP-9 as a Potential Marker for Cancer: Recent Discoveries

Cancer biomarker research is a significant cancer research field. Cancer biomarkers can play an essential role in fields such as cancer diagnosis and prognosis, monitoring disease progression, predicting disease recurrence, monitoring and predicting treatment efficacy, and cancer screening. MMP-9 has been found to be a potential biomarker for several cancers [84,85,86,87,88]. It could be explored as a biomarker in fields, such as diagnosis, treatment efficacy monitoring and disease progression monitoring. Cancer biomarker represents one promising application of MMP-9 research. Some biomarkers may lack sufficient specificity for clinical utility when they are used as a single marker. Using a combination of biomarkers is one strategy to increase the specificity of biomarkers. To achieve high specificity, MMP-9 can also be used in combination with other cancer biomarkers. In recent years, there has been much progress in cancer biomarker research about exploring MMP-9 as a biomarker for different types of cancer. Here, some recent research is summarized.

### 2.1. MMP-9 as a Potential Cancer Biomarker in Giant Cell Tumor of Bone (GCTB)

Giant-cell tumor of bone (GCTB) is an aggressive primary bone tumor. Giant-cell tumor stromal cell (GCTSC) is the tumor cell of this type of tumor. Recent research detected expression of MMP-9 in GCTSC in peripheral tissue and central tissue of GCTB [89]. In peripheral tissue, GCTSC usually displayed high MMP-9 protein levels in immunohistochemical staining assay and high mRNA expression. In the immunohistochemical analysis of this study, both pro-MMP-9 and active MMP-9 were detected. In central tissue, the MMP-9 staining grade of the multi-nucleated giant cell was high. This novel discovery indicates that MMP-9 is a potential biomarker of GCTB. Knowledge about biomarkers of GCTSC is vital for further research of GCTB.

### 2.2. MMP-9 as a Potential Cancer Biomarker in Non-Small Cell Lung Cancer (NSCLC)

Non-small cell lung cancer (NSCLC) accounts for most of all lung cancers. Although several important biomarkers, such as epidermal growth factor receptor (EGFR) mutation, have already been discovered, it is important to discover novel biomarkers of NSCLC to improve NSCLC detection [90,91].

One study evaluated the level of several matrix metalloproteases (MMPs) in an initial study cohort [92]. The authors compared the serum MMP-9 level between patient group and healthy group (19 NSCLC cases and 19 healthy controls) by multiplexed immunoassays [92]. Both pro-MMP-9 and active MMP-9 were detected in the immunoassay of this study. MMP-9 was found to be elevated in the serum of NSCLC patients compared to healthy controls. Moreover, after further evaluation using a larger sample set, the authors found that MMP-9 was a potential biomarker of NSCLC [92]. The optimal diagnostic value of MMP-9 in a model which includes MMP-9, gender, age and smoking history was also calculated [92]. This study indicates that MMP-9 is a potential biomarker for NSCLC diagnosis. Additionally, after combination with other biomarkers of NSCLC, MMP-9 can be a potential biomarker of NSCLC with significant value.

### 2.3. MMP-9 as a Potential Cancer Biomarker in Cervical Cancer

Cervical cancer is cancer that occurs in the cells of the cervix. This cancer is usually closely related to human papillomavirus (HPV) infection. One study investigated the expression of MMP-9 in cervical cancer specimens from 225 cases using immunochemistry assays [93]. Both pro-MMP-9 and active MMP-9 may be detected in this assay. MMP-9 expression was found to be elevated in cervical cancer. Patients with cervical cancer of positive MMP-9 staining tend to have worse overall survival. This study indicates that MMP-9 expression in cervical cancer is an independent prognostic factor. In several other studies, MMP-9 level in plasma was measured and compared between patient group and control groups using enzyme-linked immunosorbent assay (ELISA) [94,95,96]. These studies found that MMP-9 could be a useful biomarker in the diagnosis of cervical cancer in combination with other biomarkers.

### 2.4. MMP-9 as a Potential Cancer Biomarker in Ovarian Cancer

Ovarian cancer, which is the leading cause of gynecologic cancer mortality, is one of most commonly diagnosed cancer among women in the world [97,98]. High-grade serous ovarian cancer (HGSOC) is the most aggressive type of ovarian cancer [99]. MMP-9 has recently been found to be a biomarker of ovarian cancer [87,88]. A recent study about biomarkers of HGSOC showed that inactive pro-MMP-9 in Annexin V-binding extracellular vesicles (EV) was a putative HGSOC biomarker [99]. In this study, the authors isolated different extracellular vesicle types from the ascites of ovarian cancer patients and a control group (patients with cirrhosis). They found that MMP-9 level was higher in Annexin V-binding EVs of malignant samples compared to portal-hypertensive ascites. This research indicates that inactive pro-MMP-9 in Annexin V-binding EVs could be an HGSOC biomarker.

### 2.5. MMP-9 as a Potential Cancer Biomarker in Pancreatic Cancer

A recent study identified MMP-9 as an elevated expression protein in pancreatic juice from pancreatic ductal adenocarcinoma (PDAC) patients compared with that of healthy controls using an experimental design based on proteomic analysis and tandem mass spectrometry [100]. This discovery was confirmed by western blot assay and immunohistochemical study. In this research, both pro-MMP-9 and active MMP-9 were detected in immunohistochemical assay and western blot assay. Besides, serum MMP-9 levels measured by ELISA were found to be significantly higher in PDAC patients than those in healthy controls or patients of another control group (chronic pancreatitis patient group) [100]. This research showed that MMP-9 is a potential biomarker for pancreatic cancer.

### 2.6. MMP-9 as a Potential Cancer Biomarker in Osteosarcoma

A recent meta-analysis study evaluated the diagnostic value of MMP-9 as a biomarker to osteosarcoma [101]. It indicated that MMP-9 could be a potential biomarker for osteosarcoma. In this meta-analysis, pooled sensitivity, pooled specificity, the diagnostic odds ratio (DOR) and the area under the receiver operating characteristic curve (AUC) were calculated. In this analysis, pooled sensitivity, pooled specificity and DOR of MMP-9 for osteosarcoma were found to be 0.78 (95% confidence interval CI: 0.730–0.83), 0.90 (95% CI: 0.79–0.95) and 31.20 (95% CI: 11.97–81.31), respectively [101]. AUC, which can assess the discriminating ability of biomarkers, was found to be 0.87 (95% CI: 0.83–0.89). Substantial heterogeneity exists in this study. Another recent meta-analysis evaluated the prognostic significance of MMP-9 expression for osteosarcoma risk, and found that MMP-9 expression was associated with an increased mortality rate of osteosarcoma during the follow-up research [102]. The risk ratio (RR), its 95% confidence intervaland the *p*-value of pooled RR Z-test (risk ratio = 2.79, 95% CI: 1.96–3.97, *p* < 0.00001) were determined in this study [102].

### 2.7. MMP-9 as a Potential Cancer Biomarker in Breast Cancer

Many studies have explored the value of MMP-9 as a biomarker for breast cancer. Some of these studies are highlighted here.

In a previous study, one group of researchers investigated MMP-9 expression in normal human breast tissue and in breast cancer tissue of different molecular subtypes [103]. This study showed that MMP-9 expression in healthy breast tissue is low. It found a significant increase in MMP-9 expression in breast cancer cells. This result is consistent with some other research [104,105,106]. In the immunohistochemical analysis of this study, both pro-MMP-9 and active MMP-9 were detected [103]. Additionally, the study found that MMP-9 was differentially expressed within different molecular subtypes of breast cancer. Overexpression of MMP-9 is a clear feature of triple-negative and HER2-positive breast cancers [103]. Overexpression of MMP-9 was also found in metastatic lymph nodes. In another recent study, researchers found that, in breast cancer tissues, MMP-9 and MMP-2 expression levels were correlated with lymph node metastasis and tumor staging [106]. Both pro-MMP-9 and active MMP-9 were detected in the immunohistochemical analysis of this study [106].

Another recent study investigated the possibility of utilizing MMP-9 and RhoA level for breast cancer risk assessment through patient stratification [107]. Using active MMP-9 activity levels in serum and RhoA expression patterns in circulating leucocytes, the authors developed a non-invasive multiparametric approach to stratify patients with breast cancer. The results showed that this stratification approach using MMP-9/RhoA biomarker-combination might be useful for early breast cancer diagnostics, although the authors admitted the existence of limitations in the stratification system.

Other research investigated multiple biomarkers in metastatic breast cancer patients, including serum neuron-specific enolase (NSE) level, serum MMP-9 level, and serum HER2 extracellular domain level (ECD) [108]. MMP-9 level in this study included both pro-MMP-9 and active MMP-9. The correlation of these biomarkers with the presence of brain metastasis in metastatic breast cancer patients was evaluated. The samples group was found to contain serum from breast cancer with brain metastases, and the control group to contain serum from metastatic breast cancer patients without central nervous system involvement. This study showed that serum NSE, MMP-9, and HER2 ECD levels were significantly higher in patients with brain metastasis. In multivariate analysis, metastatic breast cancer patients with brain metastases can be accurately discriminated by serum HER2 ECD levels and serum MMP-9 levels from those patients without brain metastases.

### 2.8. MMP-9/MMP-2 Ratio as a Potential Cancer Biomarker in Hepatitis B Virus-Related Hepatocellular Carcinoma

In a recent study, researchers measured MMP-9/MMP-2 ratios in serum samples from chronic hepatitis B patients by zymography. Serum samples used in this study were from 52 healthy carriers, 47 chronic hepatitis patients, 50 cirrhosis patients and 32 hepatitis B virus-related hepatocellular carcinoma patients [109]. The authors observed that serum MMP-9/MMP-2 ratios in hepatitis B virus-related hepatocellular carcinoma patients were significantly higher than those of other groups (healthy carriers, chronic hepatitis patients and cirrhosis patients). The sensitivity and specificity of this evaluation was also calculated. Additionally, this research found that MMP-9/MMP-2 ratios in advanced, inoperable hepatitis B virus-related hepatocellular carcinoma patients were significantly higher than those in early-stage hepatitis B virus-related hepatocellular carcinoma patients. This research indicates that serum MMP-9/MMP-2 ratio is a potential biomarker of hepatitis B virus-related hepatocellular carcinoma.

### 2.9. MMP-9/NGAL Complex as a Potential Cancer Biomarker

MMP-9 and neutrophil gelatinase associated lipocalin (NGAL) proteins can form MMP-9/NGAL complex through disulfide bridges [75,110,111]. NGAL, which is a member of lipocalin superfamily protein, plays a role in innate immunity during bacterial infections, and is a promising biomarker for kidney diseases [111,112,113,114,115]. Formation of MMP-9/NGAL complex increases MMP-9 stability by protecting it from degradation. Some research already explored MMP-9/NGAL complex as a potential biomarker in some cancers [116,117,118]. For instance, one study showed that urinary MMP-9/NGAL complex was a potential noninvasive biomarker for gastric cancer, while another study found that MMP-9/NGAL activity was a potential biomarker in glioma [117,118]. MMP-9/NGAL complex is a promising biomarker because it may have relatively high specificity. The development of biosensors which can specifically detect MMP-9/NGAL complex is vital to this fields.

## 3. Recent Advances in MMP-9 Biosensor Research

Although some methods (such as ELISA) can detect MMP-9, the development of MMP-9 biosensors with high sensitivity, specificity, selectivity and compatibility is necessary. Some recent studies developed several novel MMP-9 biosensors which can improve MMP-9 detection. For some MMP-9 biosensors, the design strategy based on the knowledge that activated MMP-9 could recognize specific MMP-9 cleavage site within peptide or protein to conduct proteolytic cleavage. Among other strategies, peptide-cleavage based biosensors are one crucial group of MMP-9 biosensors.

### 3.1. Cleavage-Based MMP-9 Biosensors

**Figure 4:**
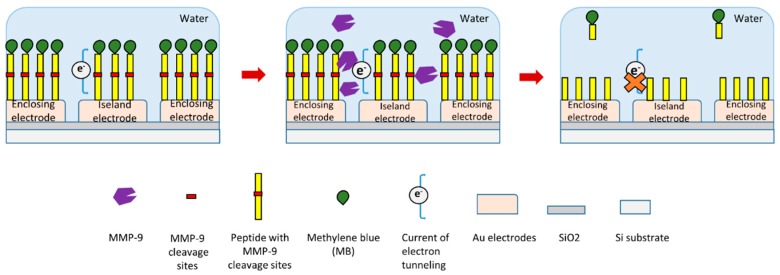
Schematic illustration of a novel peptide-cleavage based electrochemical MMP-9 biosensor [119].

In a recent study, researchers produced an electrode-free electrochemical biosensor to detect MMP-9 (Figure 4) [119]. In this study, a redox reporter labeled peptide with MMP-9 cleavage site was used. Methylene blue (MB) was conjugated at the N-terminal of this peptide. This peptide was immobilized on Au electrodes through a thiolate bond at its C-terminal (Figure 4) [119]. In the presence of MMP-9, the specific substrate peptide of MMP-9 was cleaved by it. This peptide cleavage will release electroactive MB-peptide fragment from the electrodes which can influence electrical tunneling current, which itself can be measured to detect MMP-9 (Figure 4). This biosensor can detect relatively low concentrations of MMP-9 down to about 7 pM.

**Figure 5:**
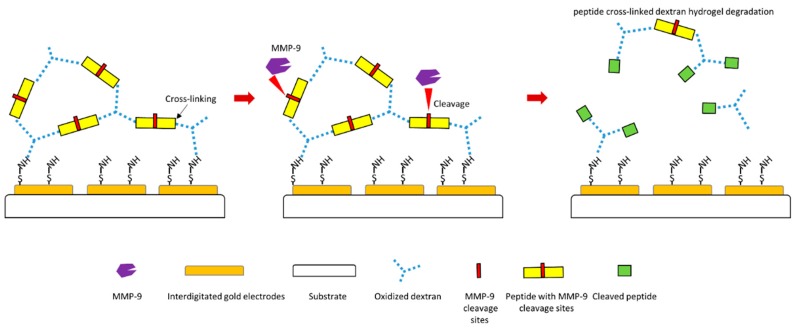
Schematic illustration of a novel peptide-cleavage based MMP-9 biosensor [120].

Another study designed a MMP-9 biosensor using hydrogel film [120]. In this biosensor design, electrodes were firstly coated with oxidized dextran, and specific peptide substrate of MMP-9 containing specific MMP-9 cleavage site was then cross-linked to dextran to form hydrogel layer (Figure 5). Therefore, if MMP-9 causes cleavage of peptide substrate at the MMP-9 cleavage site, the hydrogel layer will be degraded, which will generate a signal of this biosensor (Figure 5). This biosensor displays a negative response to MMP-2, which indicates high specificity to MMP-9 detection. This design makes MMP-9 detection much faster and more straightforward.

**Figure 6:**
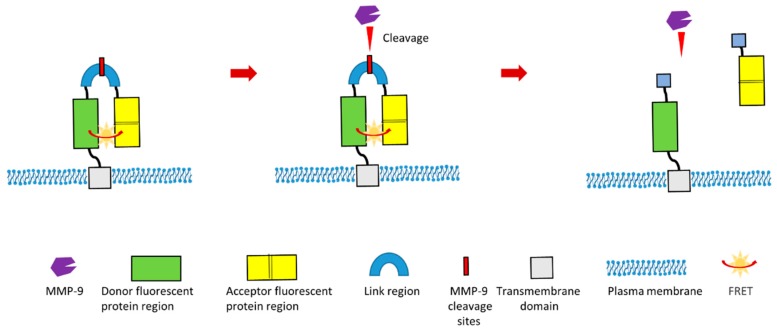
Schematic illustration of a novel cleavage-based FRET MMP-9 biosensor [121].

In other recent study, a group designed a novel Förster Resonance Energy Transfer (FRET)-based MMP-9 activity biosensor which could continuously monitor MMP-9 activity in vivo [121]. This biosensor is a chimeric protein which contains donor fluorescent protein (teal fluorescent protein) region, acceptor fluorescent protein (two tandem venus proteins) region and linker region. This linker region contains specific MMP-9 cleavage site (Figure 6). Additionally, this biosensor contains a signal peptide and a transmembrane domain. Therefore, this chimeric protein can be displayed and anchored in the plasma membrane (Figure 6). This biosensor was optimized to ensure the highest FRET efficiency before the cleavage of linker region by MMP-9. In the presence of MMP-9, this specific MMP-9 cleavage site within the linker region will be cleaved. This cleavage will result in the separation of acceptor proteins region from the donor region, which will lead to the modification of the FRET signal (Figure 6). This design of MMP-9 biosensor can be used for the in vivo imaging of MMP-9 activity, which is especially valuable for investigating the biological role of MMP-9 during pathological processes such as tumor development, cancer metastasis and cancer angiogenesis. Additionally, this biosensor gives readouts of activity with high spatial resolution.

**Figure 7:**
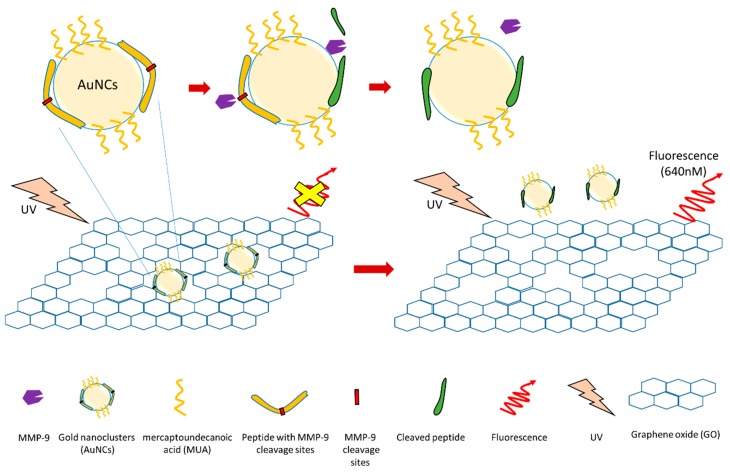
Schematic illustration of a MMP-9 biosensor based on nanotechnology [122].

Several nanotechnology-based MMP-9 biosensors have been generated recently. For example, one study developed an MMP-9 biosensor based on nanocluster/graphene nanocomplex (Figure 7) [122]. In this study, gold nanoclusters (AuNCs) were firstly synthesized using a peptide (with a MMP-9 cleavage site) and mercaptoundecanoic acid (MUA) as co-templating ligands. These AuNCs were then absorbed by graphene oxide (GO) surface to generate a peptide-MUA/AuNC/GO nanocomplex (pMAG) [122]. Graphene oxide can quench the AuNC fluorescence. During detection, active MMP-9 can cleave peptide of this nanocomplex (pMAG) at the specific MMP-9 cleavage site. This peptide cleavage will cause the dequenching of AuNC fluorescence. By detecting dequenched fluorescence, this biosensor can detect MMP-9 with high sensitivity and selectivity. Additionally, the authors used this biosensor to detect MMP-9 secreted from human adenocarcinoma cancer cells (MCF-7cells) to test its sensitivity. The detection limit of this biosensor is 2.5 ng/mL, and it only detects active MMP-9. The inhibition of MMP-9 activity causes a reduced signal.

Another study developed a nanodiamond-based MMP-9 biosensor [123]. In this biosensor, a fluorescent-labeled peptide with MMP-9 cleavage site is bonded to nanodiamond. With the presence of active MMP-9, this peptide will be cleaved. Cleavage will cause changes in fluorescence signal which can be detected during measurement. This biosensor has high selectivity and sensitivity.

### 3.2. Non-Cleavage-Based MMP-9 Biosensors

**Figure 8:**
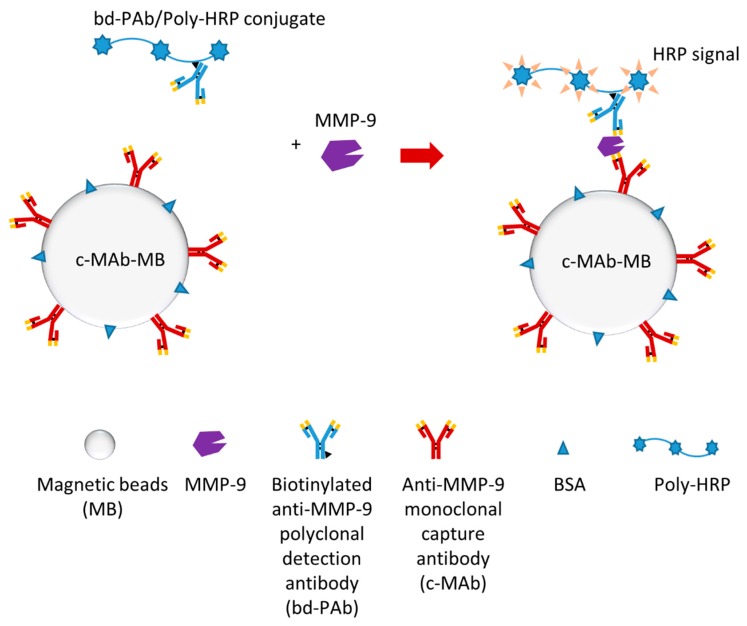
Schematic illustration of a MMP-9 electrochemical immunosensor [124].

One recent study developed an electrochemical magneto-immunosensor which could rapidly detect plasma MMP-9 (in less than 15 min) [124]. The researchers produced an immuno-modified Poly-HRP conjugate (bd-PAb/Poly-HRP) as an immuno-modified Poly-HRP signal amplifier, which was the key for this single-step magneto-immunoassay (Figure 8). Additionally, electrochemical detection was also optimized to finish detection efficiently. This biosensor can detect MMP-9 much faster and more straightforwardly compared to regular ELISA. Furthermore, this biosensor may detect both pro-MMP-9 and activate MMP-9.

**Figure 9:**
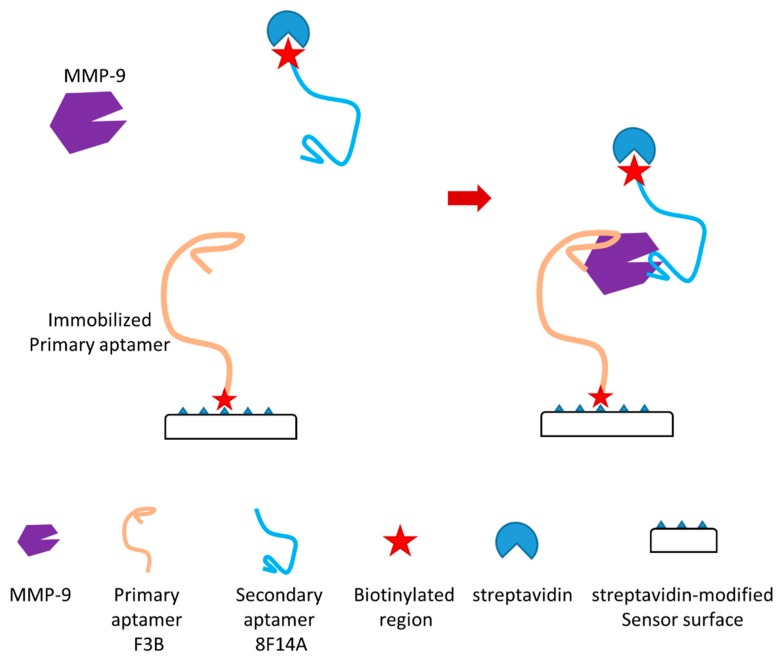
Schematic illustration of a novel aptamer-based MMP-9 biosensor [125].

In another recent study, the authors reported a novel aptamer-based MMP-9 biosensor (Figure 9) [125]. This biosensor is a dual aptamer-based piezoelectric biosensor which can detect the active form of MMP-9 with very high sensitivity. In the study, the authors showed that the detection limit in a buffer and untreated sera were 1.2 pM and 6.8 pM, respectively. Two different aptamers which can bind to different regions of MMP-9 without competition and unspecific interaction were used in this dual aptamer-based biosensor with a sandwich-like approach (Figure 9). Primary aptamer (aptamer F3B) was immobilized in the sensor surface through thiols/carboxylated dextran chemistry which can immobilize biotinylated sequences through streptavidin binding (Figure 9). Secondary aptamer (aptamer 8F14A), which can target MMP-9 on a second epitope, was utilized as a signal enhancer. The enhancement may be due to the supra-molecular architecture formed on the surface of this sensor during detection. This architecture may lead to rapid improvement of the recorded response. A quartz crystal microbalance (QCM) was applied to achieve piezoelectric transduction for this biosensor. MMP-9 detection using commercial human sera as samples was also conducted as a proof of concept. This dual aptamer-based biosensor was able to efficiently avoid immunoglobulin G (IgG) interference in this evaluation. It can detect active MMP-9 in these samples. In this study, both the specificity and sensitivity of the dual aptamer-based MMP-9 biosensor were demonstrated.

**Figure 10:**
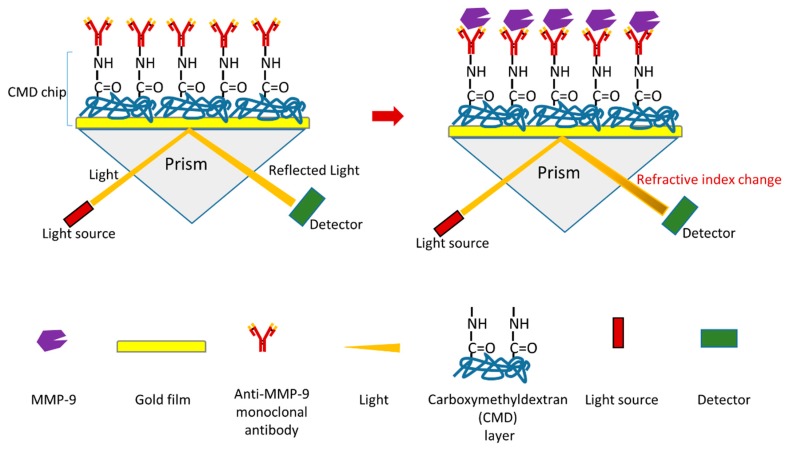
Schematic illustration of a novel SPR-based biosensor of MMP-9 [126].

A novel surface plasmon resonance (SPR) immunosensor of MMP-9 was recently reported (Figure 10) [126]. One advantage of SPR immunosensor is that it allows real-time and label-free detection. Similar to most SPR immunosensors, the mechanism of this biosensor is based on antigen–antibody interactions (Figure 10) [126]. In this research, a monoclonal anti-MMP-9 antibody was firstly immobilized via its primary amine groups onto carboxymethyldextran (CMD) chip surface using a covalent coupling method to generate a sensor chip. During MMP-9 detection, the interaction between MMP-9 and monoclonal anti-MMP-9 antibody will influence the refractive index. MMP-9 can be detected by measuring the changes of refractive index using a SPR device. The detection limit of this MMP-9 biosensor is 8 pg/mL. This biosensor can detect both pro-MMP-9 and active MMP-9. This research also used this biosensor to measure MMP-9 in saliva samples (from chronic periodontitis patients and healthy controls) as a proof of concept. The study shows that this biosensor can detect MMP-9 with a high reproducibility and sensitivity. Furthermore, this biosensor is reusable.

## 4. Conclusions and Outlook

This review summarizes some recent advances in exploring MMP-9 as a potential biomarker in cancers, and recent discoveries of novel MMP-9 biosensors are also presented. Exploring the utilization of MMP-9 as a cancer biomarker is a promising research area. MMP-9 has the potential to be clinically applied in tools in some types of cancer. MMP-9 biosensor research is a critical field for the translational application of knowledge about using MMP-9 as a biomarker.

Although MMP-9 has been found to be a potential cancer biomarker in several types of cancer, many improvements are still needed in this research area. Firstly and most importantly, to explore the translational application value of MMP-9 as a potential cancer biomarker (or as one biomarker within a biomarker combination) in one specific cancer, systematic high quality evaluations are critical. Such evaluations should usually involve a large number of individuals in patient groups and healthy groups. If a conclusion about the value of an application can be achieved through a strict evaluation, it will be a significant achievement. This kind of research, which usually requires much effort and input, is still rare. Secondly, translational research which can translate some MMP-9 biosensors with applicational value into real clinical application is fundamental. If more progress in the translational application is achieved, it will dramatically advance this field. MMP-9 inhibitors are the basis for targeting MMP-9 in some cancers and other diseases, and this field may achieve breakthroughs in the future.

## References

[1] Klein T., Bischoff R. (2011). Physiology and pathophysiology of matrix metalloproteases. Amino Acids.

[2] Nagase H., Visse R., Murphy G. (2006). Structure and function of matrix metalloproteinases and timps. Cardiovasc. Res..

[3] Vandooren J., Van den Steen P.E., Opdenakker G. (2013). Biochemistry and molecular biology of gelatinase B or matrix metalloproteinase-9 (MMP-9): The next decade. Crit. Rev. Biochem. Mol. Biol..

[4] Bruschi F., Pinto B. (2013). The significance of matrix metalloproteinases in parasitic infections involving the central nervous system. Pathogens.

[5] Van den Steen P.E., Dubois B., Nelissen I., Rudd P.M., Dwek R.A., Opdenakker G. (2002). Biochemistry and molecular biology of gelatinase B or matrix metalloproteinase-9 (MMP-9). Crit. Rev. Biochem. Mol. Biol..

[6] Agrawal A., Romero-Perez D., Jacobsen J.A., Villarreal F.J., Cohen S.M. (2008). Zinc-binding groups modulate selective inhibition of MMPs. ChemMedChem.

[7] Santos M.C., de Souza A.P., Gerlach R.F., Trevilatto P.C., Scarel-Caminaga R.M., Line S.R. (2004). Inhibition of human pulpal gelatinases (MMP-2 and MMP-9) by zinc oxide cements. J. Oral Rehabil..

[8] Opdenakker G., Van den Steen P.E., Dubois B., Nelissen I., Van Coillie E., Masure S., Proost P., Van Damme J. (2001). Gelatinase B functions as regulator and effector in leukocyte biology. J. Leukoc. Biol..

[9] Rosenblum G., Van den Steen P.E., Cohen S.R., Grossmann J.G., Frenkel J., Sertchook R., Slack N., Strange R.W., Opdenakker G., Sagi I. (2007). Insights into the structure and domain flexibility of full-length pro-matrix metalloproteinase-9/gelatinase B. Structure.

[10] Overall C.M., Butler G.S. (2007). Protease yoga: Extreme flexibility of a matrix metalloproteinase. Structure.

[11] Shipley J.M., Doyle G.A., Fliszar C.J., Ye Q.Z., Johnson L.L., Shapiro S.D., Welgus H.G., Senior R.M. (1996). The structural basis for the elastolytic activity of the 92-kDa and 72-kDa gelatinases. Role of the fibronectin type ii-like repeats. J. Biol. Chem..

[12] O’Farrell T.J., Pourmotabbed T. (1998). The fibronectin-like domain is required for the type V and XI collagenolytic activity of gelatinase B. Arch. Biochem. Biophys..

[13] Lauer-Fields J.L., Whitehead J.K., Li S., Hammer R.P., Brew K., Fields G.B. (2008). Selective modulation of matrix metalloproteinase 9 (MMP-9) functions via exosite inhibition. J. Biol. Chem..

[14] Roeb E., Schleinkofer K., Kernebeck T., Potsch S., Jansen B., Behrmann I., Matern S., Grotzinger J. (2002). The matrix metalloproteinase 9 (MMP-9) hemopexin domain is a novel gelatin binding domain and acts as an antagonist. J. Biol. Chem..

[15] Ethell I.M., Ethell D.W. (2007). Matrix metalloproteinases in brain development and remodeling: Synaptic functions and targets. J. Neurosci. Res..

[16] Roderfeld M., Graf J., Giese B., Salguero-Palacios R., Tschuschner A., Muller-Newen G., Roeb E. (2007). Latent MMP-9 is bound to TIMP-1 before secretion. Biol. Chem..

[17] Yabluchanskiy A., Ma Y., Iyer R.P., Hall M.E., Lindsey M.L. (2013). Matrix metalloproteinase-9: Many shades of function in cardiovascular disease. Physiology.

[18] Massova I., Kotra L.P., Fridman R., Mobashery S. (1998). Matrix metalloproteinases: Structures, evolution, and diversification. FASEB J..

[19] Dufour A., Sampson N.S., Li J., Kuscu C., Rizzo R.C., Deleon J.L., Zhi J., Jaber N., Liu E., Zucker S. (2011). Small-molecule anticancer compounds selectively target the hemopexin domain of matrix metalloproteinase-9. Cancer Res..

[20] Reinhard S.M., Razak K., Ethell I.M. (2015). A delicate balance: Role of MMP-9 in brain development and pathophysiology of neurodevelopmental disorders. Front. Cell. Neurosci..

[21] Ogata Y., Enghild J.J., Nagase H. (1992). Matrix metalloproteinase 3 (stromelysin) activates the precursor for the human matrix metalloproteinase 9. J. Biol. Chem..

[22] Fridman R., Toth M., Pena D., Mobashery S. (1995). Activation of progelatinase b (MMP-9) by gelatinase a (MMP-2). Cancer Res..

[23] Imai K., Yokohama Y., Nakanishi I., Ohuchi E., Fujii Y., Nakai N., Okada Y. (1995). Matrix metalloproteinase 7 (matrilysin) from human rectal carcinoma cells. Activation of the precursor, interaction with other matrix metalloproteinases and enzymic properties. J. Biol. Chem..

[24] Knauper V., Smith B., Lopez-Otin C., Murphy G. (1997). Activation of progelatinase B (proMMP-9) by active collagenase-3 (MMP-13). Eur. J. Biochem..

[25] Rajagopalan S., Meng X.P., Ramasamy S., Harrison D.G., Galis Z.S. (1996). Reactive oxygen species produced by macrophage-derived foam cells regulate the activity of vascular matrix metalloproteinases in vitro. Implications for atherosclerotic plaque stability. J. Clin. Investig..

[26] Gu Z., Kaul M., Yan B., Kridel S.J., Cui J., Strongin A., Smith J.W., Liddington R.C., Lipton S.A. (2002). S-nitrosylation of matrix metalloproteinases: Signaling pathway to neuronal cell death. Science.

[27] Paquette B., Bisson M., Therriault H., Lemay R., Pare M., Banville P., Cantin A.M. (2003). Activation of matrix metalloproteinase-2 and -9 by 2- and 4-hydroxyestradiol. J. Steroid Biochem. Mol. Biol..

[28] Manabe S., Gu Z., Lipton S.A. (2005). Activation of matrix metalloproteinase-9 via neuronal nitric oxide synthase contributes to NMDA-induced retinal ganglion cell death. Investig. Ophthalmol. Vis. Sci..

[29] Ridnour L.A., Windhausen A.N., Isenberg J.S., Yeung N., Thomas D.D., Vitek M.P., Roberts D.D., Wink D.A. (2007). Nitric oxide regulates matrix metalloproteinase-9 activity by guanylyl-cyclase-dependent and -independent pathways. Proc. Natl. Acad. Sci. USA.

[30] Kridel S.J., Chen E., Kotra L.P., Howard E.W., Mobashery S., Smith J.W. (2001). Substrate hydrolysis by matrix metalloproteinase-9. J. Biol. Chem..

[31] Prudova A., Auf dem Keller U., Butler G.S., Overall C.M. (2010). Multiplex n-terminome analysis of MMP-2 and MMP-9 substrate degradomes by itraq-tails quantitative proteomics. Mol. Cell. Proteom..

[32] Fan D., Wang Y., Qi P., Chen Y., Xu P., Yang X., Jin X., Tian X. (2016). Microrna-183 functions as the tumor suppressor via inhibiting cellular invasion and metastasis by targeting MMP-9 in cervical cancer. Gynecol. Oncol..

[33] Aung L.L., Mouradian M.M., Dhib-Jalbut S., Balashov K.E. (2015). MMP-9 expression is increased in b lymphocytes during multiple sclerosis exacerbation and is regulated by microrna-320a. J. Neuroimmunol..

[34] Jiang Y., Muschel R.J. (2002). Regulation of matrix metalloproteinase-9 (MMP-9) by translational efficiency in murine prostate carcinoma cells. Cancer Res..

[35] Melamed D., Messika O., Glass-Marmor L., Miller A. (2006). Modulation of matrix metalloproteinase-9 (MMP-9) secretion in B lymphopoiesis. Int. Immunol..

[36] Ong C.W., Pabisiak P.J., Brilha S., Singh P., Roncaroli F., Elkington P.T., Friedland J.S. (2017). Complex regulation of neutrophil-derived MMP-9 secretion in central nervous system tuberculosis. J. Neuroinflamm..

[37] Li L., Li H. (2013). Role of microrna-mediated MMP regulation in the treatment and diagnosis of malignant tumors. Cancer Biol. Ther..

[38] Zariffard M.R., Anastos K., French A.L., Munyazesa E., Cohen M., Landay A.L., Spear G.T. (2015). Cleavage/alteration of interleukin-8 by matrix metalloproteinase-9 in the female lower genital tract. PLoS ONE.

[39] Backstrom J.R., Lim G.P., Cullen M.J., Tokes Z.A. (1996). Matrix metalloproteinase-9 (MMP-9) is synthesized in neurons of the human hippocampus and is capable of degrading the amyloid-beta peptide (1–40). J. Neurosci..

[40] Mohan R., Chintala S.K., Jung J.C., Villar W.V., McCabe F., Russo L.A., Lee Y., McCarthy B.E., Wollenberg K.R., Jester J.V. (2002). Matrix metalloproteinase gelatinase b (MMP-9) coordinates and effects epithelial regeneration. J. Biol. Chem..

[41] Martinez-Hernandez M.G., Baiza-Gutman L.A., Castillo-Trapala A., Armant D.R. (2011). Regulation of proteinases during mouse peri-implantation development: Urokinase-type plasminogen activator expression and cross talk with matrix metalloproteinase 9. Reproduction.

[42] Dziembowska M., Wlodarczyk J. (2012). MMP9: A novel function in synaptic plasticity. Int. J. Biochem. Cell Biol..

[43] Agrawal S.M., Lau L., Yong V.W. (2008). MMPs in the central nervous system: Where the good guys go bad. Semin. Cell Dev. Biol..

[44] Stamenkovic I. (2003). Extracellular matrix remodelling: The role of matrix metalloproteinases. J. Pathol..

[45] Farina A.R., Mackay A.R. (2014). Gelatinase b/MMP-9 in tumour pathogenesis and progression. Cancers.

[46] Fiore E., Fusco C., Romero P., Stamenkovic I. (2002). Matrix metalloproteinase 9 (MMP-9/gelatinase B) proteolytically cleaves ICAM-1 and participates in tumor cell resistance to natural killer cell-mediated cytotoxicity. Oncogene.

[47] Vaisar T., Kassim S.Y., Gomez I.G., Green P.S., Hargarten S., Gough P.J., Parks W.C., Wilson C.L., Raines E.W., Heinecke J.W. (2009). MMP-9 sheds the beta2 integrin subunit (CD18) from macrophages. Mol. Cell. Proteom. MCP.

[48] Cauwe B., Opdenakker G. (2010). Intracellular substrate cleavage: A novel dimension in the biochemistry, biology and pathology of matrix metalloproteinases. Crit. Rev. Biochem. Mol. Biol..

[49] Jobin P.G., Butler G.S., Overall C.M. (2017). New intracellular activities of matrix metalloproteinases shine in the moonlight. Biochim. Biophys. Acta.

[50] Zhang Z., Amorosa L.F., Coyle S.M., Macor M.A., Lubitz S.E., Carson J.L., Birnbaum M.J., Lee L.Y., Haimovich B. (2015). Proteolytic cleavage of ampkalpha and intracellular MMP9 expression are both required for tlr4-mediated mtorc1 activation and hif-1alpha expression in leukocytes. J. Immunol..

[51] Nguyen M., Arkell J., Jackson C.J. (1998). Active and tissue inhibitor of matrix metalloproteinase-free gelatinase b accumulates within human microvascular endothelial vesicles. J. Biol. Chem..

[52] Zhao Y.G., Xiao A.Z., Newcomer R.G., Park H.I., Kang T., Chung L.W., Swanson M.G., Zhau H.E., Kurhanewicz J., Sang Q.X. (2003). Activation of pro-gelatinase b by endometase/matrilysin-2 promotes invasion of human prostate cancer cells. J. Biol. Chem..

[53] Kowluru R.A., Mohammad G., dos Santos J.M., Zhong Q. (2011). Abrogation of MMP-9 gene protects against the development of retinopathy in diabetic mice by preventing mitochondrial damage. Diabetes.

[54] Hill J.W., Poddar R., Thompson J.F., Rosenberg G.A., Yang Y. (2012). Intranuclear matrix metalloproteinases promote DNA damage and apoptosis induced by oxygen-glucose deprivation in neurons. Neuroscience.

[55] Yang Y., Candelario-Jalil E., Thompson J.F., Cuadrado E., Estrada E.Y., Rosell A., Montaner J., Rosenberg G.A. (2010). Increased intranuclear matrix metalloproteinase activity in neurons interferes with oxidative DNA repair in focal cerebral ischemia. J. Neurochem..

[56] Hou H., Zhang G., Wang H., Gong H., Wang C., Zhang X. (2014). High matrix metalloproteinase-9 expression induces angiogenesis and basement membrane degradation in stroke-prone spontaneously hypertensive rats after cerebral infarction. Neural Regen. Res..

[57] Misko A., Ferguson T., Notterpek L. (2002). Matrix metalloproteinase mediated degradation of basement membrane proteins in trembler j neuropathy nerves. J. Neurochem..

[58] Ozdemir E., Kakehi Y., Okuno H., Yoshida O. (1999). Role of matrix metalloproteinase-9 in the basement membrane destruction of superficial urothelial carcinomas. J. Urol..

[59] Hsu C.C., Huang S.F., Wang J.S., Chu W.K., Nien J.E., Chen W.S., Chow S.E. (2016). Interplay of n-cadherin and matrix metalloproteinase 9 enhances human nasopharyngeal carcinoma cell invasion. BMC Cancer.

[60] Kim Y.H., Kwon H.J., Kim D.S. (2012). Matrix metalloproteinase 9 (MMP-9)-dependent processing of betaig-h3 protein regulates cell migration, invasion, and adhesion. J. Biol. Chem..

[61] Dwivedi A., Slater S.C., George S.J. (2009). MMP-9 and -12 cause n-cadherin shedding and thereby beta-catenin signalling and vascular smooth muscle cell proliferation. Cardiovasc. Res..

[62] Ortega N., Behonick D.J., Colnot C., Cooper D.N., Werb Z. (2005). Galectin-3 is a downstream regulator of matrix metalloproteinase-9 function during endochondral bone formation. Mol. Biol. Cell.

[63] Gialeli C., Theocharis A.D., Karamanos N.K. (2011). Roles of matrix metalloproteinases in cancer progression and their pharmacological targeting. FEBS J..

[64] Mehner C., Hockla A., Miller E., Ran S., Radisky D.C., Radisky E.S. (2014). Tumor cell-produced matrix metalloproteinase 9 (MMP-9) drives malignant progression and metastasis of basal-like triple negative breast cancer. Oncotarget.

[65] Xu D., McKee C.M., Cao Y., Ding Y., Kessler B.M., Muschel R.J. (2010). Matrix metalloproteinase-9 regulates tumor cell invasion through cleavage of protease nexin-1. Cancer Res..

[66] Pego E.R., Fernandez I., Nunez M.J. (2018). Molecular basis of the effect of MMP-9 on the prostate bone metastasis: A review. Urol. Oncol..

[67] Itoh T., Tanioka M., Matsuda H., Nishimoto H., Yoshioka T., Suzuki R., Uehira M. (1999). Experimental metastasis is suppressed in MMP-9-deficient mice. Clin. Exp. Metast..

[68] Wang X., Nagase H., Watanabe T., Nobusue H., Suzuki T., Asami Y., Shinojima Y., Kawashima H., Takagi K., Mishra R. (2010). Inhibition of MMP-9 transcription and suppression of tumor metastasis by pyrrole-imidazole polyamide. Cancer Sci..

[69] Chou C.H., Teng C.M., Tzen K.Y., Chang Y.C., Chen J.H., Cheng J.C. (2012). MMP-9 from sublethally irradiated tumor promotes lewis lung carcinoma cell invasiveness and pulmonary metastasis. Oncogene.

[70] Hawinkels L.J., Zuidwijk K., Verspaget H.W., de Jonge-Muller E.S., van Duijn W., Ferreira V., Fontijn R.D., David G., Hommes D.W., Lamers C.B. (2008). VEGF release by MMP-9 mediated heparan sulphate cleavage induces colorectal cancer angiogenesis. Eur. J. Cancer.

[71] Leifler K.S., Svensson S., Abrahamsson A., Bendrik C., Robertson J., Gauldie J., Olsson A.K., Dabrosin C. (2013). Inflammation induced by MMP-9 enhances tumor regression of experimental breast cancer. J. Immunol..

[72] Zhang Y., Chen Q. (2017). Relationship between matrix metalloproteinases and the occurrence and development of ovarian cancer. Braz. J. Med. Biol. Res..

[73] Kessenbrock K., Plaks V., Werb Z. (2010). Matrix metalloproteinases: Regulators of the tumor microenvironment. Cell.

[74] Bruni-Cardoso A., Johnson L.C., Vessella R.L., Peterson T.E., Lynch C.C. (2010). Osteoclast-derived matrix metalloproteinase-9 directly affects angiogenesis in the prostate tumor-bone microenvironment. Mol. Cancer Res..

[75] Candido S., Abrams S.L., Steelman L.S., Lertpiriyapong K., Fitzgerald T.L., Martelli A.M., Cocco L., Montalto G., Cervello M., Polesel J. (2016). Roles of ngal and MMP-9 in the tumor microenvironment and sensitivity to targeted therapy. Biochim. Biophys. Acta.

[76] Garg P., Sarma D., Jeppsson S., Patel N.R., Gewirtz A.T., Merlin D., Sitaraman S.V. (2010). Matrix metalloproteinase-9 functions as a tumor suppressor in colitis-associated cancer. Cancer Res..

[77] Pujada A., Walter L., Patel A., Bui T.A., Zhang Z., Zhang Y., Denning T.L., Garg P. (2017). Matrix metalloproteinase MMP9 maintains epithelial barrier function and preserves mucosal lining in colitis associated cancer. Oncotarget.

[78] Walter L., Pujada A., Bhatnagar N., Bialkowska A.B., Yang V.W., Laroui H., Garg P. (2017). Epithelial derived-matrix metalloproteinase (MMP9) exhibits a novel defensive role of tumor suppressor in colitis associated cancer by activating MMP9-Notch1-ARF-p53 axis. Oncotarget.

[79] Rahimi Z., Abdan Z., Rahimi Z., Razazian N., Shiri H., Vaisi-Raygani A., Shakiba E., Vessal M., Moradi M.T. (2016). Functional promoter polymorphisms of MMP-2 C-735T and MMP-9 C-1562T and their synergism with MMP-7 A-181G in multiple sclerosis. Immunol. Investig..

[80] Ram M., Sherer Y., Shoenfeld Y. (2006). Matrix metalloproteinase-9 and autoimmune diseases. J. Clin. Immunol..

[81] De Rooy D.P., Zhernakova A., Tsonaka R., Willemze A., Kurreeman B.A., Trynka G., van Toorn L., Toes R.E., Huizinga T.W., Houwing-Duistermaat J.J. (2014). A genetic variant in the region of MMP-9 is associated with serum levels and progression of joint damage in rheumatoid arthritis. Ann. Rheum. Dis..

[82] Xue M., McKelvey K., Shen K., Minhas N., March L., Park S.Y., Jackson C.J. (2014). Endogenous MMP-9 and not MMP-2 promotes rheumatoid synovial fibroblast survival, inflammation and cartilage degradation. Rheumatology.

[83] Naouali A., Kaabachi W., Tizaoui K., Amor A.B., Hamzaoui A., Hamzaoui K. (2015). Association of MMP-9 gene polymorphisms with behcet’s disease risk. Immunol. Lett..

[84] Liang S., Chang L. (2018). Serum matrix metalloproteinase-9 level as a biomarker for colorectal cancer: A diagnostic meta-analysis. Biomark. Med..

[85] Shao W., Wang W., Xiong X.G., Cao C., Yan T.D., Chen G., Chen H., Yin W., Liu J., Gu Y. (2011). Prognostic impact of MMP-2 and MMP-9 expression in pathologic stage ia non-small cell lung cancer. J. Surg. Oncol..

[86] Roy R., Yang J., Moses M.A. (2009). Matrix metalloproteinases as novel biomarkers and potential therapeutic targets in human cancer. J. Clin. Oncol..

[87] Li L.N., Zhou X., Gu Y., Yan J. (2013). Prognostic value of MMP-9 in ovarian cancer: A meta-analysis. Asian Pac. J. Cancer Prev..

[88] Hu X., Li D., Zhang W., Zhou J., Tang B., Li L. (2012). Matrix metalloproteinase-9 expression correlates with prognosis and involved in ovarian cancer cell invasion. Arch. Gynecol. Obstet..

[89] Chen L., Zhang J., He Y., Ding X.Y. (2018). Matrix metalloproteinase-9 expression of gctsc in peripheral tissue and central tissue of gctb. J. Cell. Biochem..

[90] Burotto M., Thomas A., Subramaniam D., Giaccone G., Rajan A. (2014). Biomarkers in early-stage non-small-cell lung cancer: Current concepts and future directions. J. Thorac. Oncol..

[91] Korpanty G.J., Graham D.M., Vincent M.D., Leighl N.B. (2014). Biomarkers that currently affect clinical practice in lung cancer: Egfr, alk, met, ros-1, and kras. Front. Oncol..

[92] Blanco-Prieto S., Barcia-Castro L., Paez de la Cadena M., Rodriguez-Berrocal F.J., Vazquez-Iglesias L., Botana-Rial M.I., Fernandez-Villar A., De Chiara L. (2017). Relevance of matrix metalloproteases in non-small cell lung cancer diagnosis. BMC Cancer.

[93] Li Y., Wu T., Zhang B., Yao Y., Yin G. (2012). Matrix metalloproteinase-9 is a prognostic marker for patients with cervical cancer. Med. Oncol..

[94] Zajkowska M., Zbucka-Kretowska M., Sidorkiewicz I., Lubowicka E., Bedkowska G.E., Gacuta E., Szmitkowski M., Lawicki S. (2018). Human Plasma Levels of Vascular Endothelial Growth Factor, Matrix Metalloproteinase 9, and Tissue Inhibitor of Matrix Metalloproteinase 1 and Their Applicability as Tumor Markers in Diagnoses of Cervical Cancer Based on ROC Analysis. Cancer Control J. Moffitt Cancer Cent..

[95] Zajkowska M., Zbucka-Kretowska M., Sidorkiewicz I., Lubowicka E., Gacuta E., Szmitkowski M., Chrostek L., Lawicki S. (2018). Plasma levels and diagnostic utility of macrophage-colony stimulating factor, matrix metalloproteinase-9 and tissue inhibitor of metalloproteinase-1 as tumor markers in cervical cancer patients. Tumour Biol. J. Int. Soc. Oncodev. Biol. Med..

[96] Lubowicka E., Gacuta E., Zajkowska M., Glazewska E.K., Przylipiak A., Chrostek L., Zbucka-Kretowska M., Lawicki S. (2017). [The plasma levels and diagnostic utility of matrix metalloproteinase-9 and CA 125 in cervical cancer patients]. Pol. Merkur. Lekarski.

[97] Matulonis U.A., Sood A.K., Fallowfield L., Howitt B.E., Sehouli J., Karlan B.Y. (2016). Ovarian cancer. Nat. Rev. Dis. Prim..

[98] Reid B.M., Permuth J.B., Sellers T.A. (2017). Epidemiology of ovarian cancer: A review. Cancer Biol. Med..

[99] Reiner A.T., Tan S., Agreiter C., Auer K., Bachmayr-Heyda A., Aust S., Pecha N., Mandorfer M., Pils D., Brisson A.R. (2017). Ev-associated MMP9 in high-grade serous ovarian cancer is preferentially localized to annexin v-binding evs. Dis. Mark..

[100] Tian M., Cui Y.Z., Song G.H., Zong M.J., Zhou X.Y., Chen Y., Han J.X. (2008). Proteomic analysis identifies MMP-9, DJ-1 and A1BG as overexpressed proteins in pancreatic juice from pancreatic ductal adenocarcinoma patients. BMC Cancer.

[101] Wang J., Shi Q., Yuan T.X., Song Q.L., Zhang Y., Wei Q., Zhou L., Luo J., Zuo G., Tang M. (2014). Matrix metalloproteinase 9 (MMP-9) in osteosarcoma: Review and meta-analysis. Clin. Chim. Acta Int. J. Clin. Chem..

[102] Liu Y., Wang Y., Teng Z., Chen J., Li Y., Chen Z., Li Z., Zhang Z. (2017). Matrix metalloproteinase 9 expression and survival of patients with osteosarcoma: A meta-analysis. Eur. J. Cancer Care.

[103] Yousef E.M., Tahir M.R., St-Pierre Y., Gaboury L.A. (2014). MMP-9 expression varies according to molecular subtypes of breast cancer. BMC Cancer.

[104] Cao D., Polyak K., Halushka M.K., Nassar H., Kouprina N., Iacobuzio-Donahue C., Wu X., Sukumar S., Hicks J., De Marzo A. (2008). Serial analysis of gene expression of lobular carcinoma in situ identifies down regulation of claudin 4 and overexpression of matrix metalloproteinase 9. Breast Cancer Res..

[105] Roomi M.W., Monterrey J.C., Kalinovsky T., Rath M., Niedzwiecki A. (2009). Distinct patterns of matrix metalloproteinase-2 and -9 expression in normal human cell lines. Oncol. Rep..

[106] Li H., Qiu Z., Li F., Wang C. (2017). The relationship between MMP-2 and MMP-9 expression levels with breast cancer incidence and prognosis. Oncol. Lett..

[107] Golubnitschaja O., Yeghiazaryan K., Abraham J.A., Schild H.H., Costigliola V., Debald M., Kuhn W. (2017). Breast cancer risk assessment: A non-invasive multiparametric approach to stratify patients by MMP-9 serum activity and RhoA expression patterns in circulating leucocytes. Amino Acids.

[108] Darlix A., Lamy P.J., Lopez-Crapez E., Braccini A.L., Firmin N., Romieu G., Thezenas S., Jacot W. (2016). Serum NSE, MMP-9 and HER2 extracellular domain are associated with brain metastases in metastatic breast cancer patients: Predictive biomarkers for brain metastases?. Int. J. Cancer.

[109] Yeh H.C., Lin S.M., Chen M.F., Pan T.L., Wang P.W., Yeh C.T. (2010). Evaluation of serum matrix metalloproteinase (MMP)-9 to MMP-2 ratio as a biomarker in hepatocellular carcinoma. Hepato-Gastroenterology.

[110] Yan L., Borregaard N., Kjeldsen L., Moses M.A. (2001). The high molecular weight urinary matrix metalloproteinase (MMP) activity is a complex of gelatinase B/MMP-9 and neutrophil gelatinase-associated lipocalin (NGAL). Modulation of MMP-9 activity by NGAL. J. Biol. Chem..

[111] Chakraborty S., Kaur S., Guha S., Batra S.K. (2012). The multifaceted roles of neutrophil gelatinase associated lipocalin (NGAL) in inflammation and cancer. Biochim. Biophys. Acta.

[112] Haase M., Bellomo R., Devarajan P., Schlattmann P., Haase-Fielitz A., NGAL Meta-analysis Investigator Group (2009). Accuracy of neutrophil gelatinase-associated lipocalin (NGAL) in diagnosis and prognosis in acute kidney injury: A systematic review and meta-analysis. Am. J. Kidney Dis..

[113] Devarajan P. (2010). Review: Neutrophil gelatinase-associated lipocalin: A troponin-like biomarker for human acute kidney injury. Nephrology.

[114] Shemin D., Dworkin L.D. (2011). Neutrophil gelatinase-associated lipocalin (NGAL) as a biomarker for early acute kidney injury. Crit. Care Clin..

[115] Mitsnefes M.M., Kathman T.S., Mishra J., Kartal J., Khoury P.R., Nickolas T.L., Barasch J., Devarajan P. (2007). Serum neutrophil gelatinase-associated lipocalin as a marker of renal function in children with chronic kidney disease. Pediatr. Nephrol..

[116] Fernandez C.A., Yan L., Louis G., Yang J., Kutok J.L., Moses M.A. (2005). The matrix metalloproteinase-9/neutrophil gelatinase-associated lipocalin complex plays a role in breast tumor growth and is present in the urine of breast cancer patients. Clin. Cancer Res..

[117] Liu M.F., Hu Y.Y., Jin T., Xu K., Wang S.H., Du G.Z., Wu B.L., Li L.Y., Xu L.Y., Li E.M. (2015). Matrix metalloproteinase-9/neutrophil gelatinase-associated lipocalin complex activity in human glioma samples predicts tumor presence and clinical prognosis. Dis. Mark..

[118] Shimura T., Dagher A., Sachdev M., Ebi M., Yamada T., Yamada T., Joh T., Moses M.A. (2015). Urinary adam12 and MMP-9/NGAL complex detect the presence of gastric cancer. Cancer Prev. Res..

[119] Lee J., Yun J.Y., Lee W.C., Choi S., Lim J., Jeong H., Shin D.-S., Park Y.J. (2017). A reference electrode-free electrochemical biosensor for detecting MMP-9 using a concentric electrode device. Sens. Actuators B Chem..

[120] Biela A., Watkinson M., Meier U.C., Baker D., Giovannoni G., Becer C.R., Krause S. (2015). Disposable MMP-9 sensor based on the degradation of peptide cross-linked hydrogel films using electrochemical impedance spectroscopy. Biosens. Bioelectron..

[121] Stawarski M., Rutkowska-Wlodarczyk I., Zeug A., Bijata M., Madej H., Kaczmarek L., Wlodarczyk J. (2014). Genetically encoded fret-based biosensor for imaging MMP-9 activity. Biomaterials.

[122] Nguyen P.D., Cong V.T., Baek C., Min J. (2017). Fabrication of peptide stabilized fluorescent gold nanocluster/graphene oxide nanocomplex and its application in turn-on detection of metalloproteinase-9. Biosens. Bioelectron..

[123] Wang X., Gu M., Toh T.B., Abdullah N.L.B., Chow E.K. (2018). Stimuli-responsive nanodiamond-based biosensor for enhanced metastatic tumor site detection. SLAS Technol..

[124] Ruiz-Vega G., Garcia-Robaina A., Ben Ismail M., Pasamar H., Garcia-Berrocoso T., Montaner J., Zourob M., Othmane A., Del Campo F.J., Baldrich E. (2018). Detection of plasma MMP-9 within minutes. Unveiling some of the clues to develop fast and simple electrochemical magneto-immunosensors. Biosens. Bioelectron..

[125] Scarano S., Dausse E., Crispo F., Toulme J.J., Minunni M. (2015). Design of a dual aptamer-based recognition strategy for human matrix metalloproteinase 9 protein by piezoelectric biosensors. Anal. Chim. Acta.

[126] Mohseni S., Moghadam T.T., Dabirmanesh B., Jabbari S., Khajeh K. (2016). Development of a label-free spr sensor for detection of matrixmetalloproteinase-9 by antibody immobilization on carboxymethyldextran chip. Biosens. Bioelectron..

